# A paradox of problems in accessing general practice: a qualitative participatory case study

**DOI:** 10.3399/BJGP.2023.0276

**Published:** 2024-01-23

**Authors:** Jennifer Voorhees, Simon Bailey, Heather Waterman, Kath Checkland

**Affiliations:** Centre for Primary Care and Health Services Research, University of Manchester, Manchester.; Centre for Health Services Studies, University of Kent, Kent.; Formerly School of Healthcare Sciences, Cardiff University, Cardiff.; Centre for Primary Care and Health Services Research, University of Manchester, Manchester.

**Keywords:** access to health care, continuity of care, general practice, health inequalities, health policy, service organisation

## Abstract

**Background:**

Despite longstanding problems of access to general practice, attempts to understand and address the issues do not adequately include perspectives of the people providing or using care, nor do they use established theories of access to understand complexity.

**Aim:**

To understand problems of access to general practice from the multiple perspectives of service users and staff using an applied theory of access.

**Design and setting:**

A qualitative participatory case study in an area of northwest England.

**Method:**

A community-based participatory approach was used with qualitative interviews, focus groups, and observation to understand perspectives about accessing general practice. Data were collected between October 2015 and October 2016. Inductive and abductive analysis, informed by Levesque *et al*’s theory of access, allowed the team to identify complexities and relationships between interrelated problems.

**Results:**

This study presents a paradox of problems in accessing general practice, in which the demand on general practice both creates and hides unmet need in the population. Data show how reactive rules to control demand have undermined important aspects of care, such as continuity. The layers of rules and decreased continuity create extra work for practice staff, clinicians, and patients. Complicated rules, combined with a lack of capacity to reach out or be flexible, leave many patients, including those with complex and/or unrecognised health needs, unable to navigate the system to access care. This relationship between demand and unmet need exacerbates existing health inequities.

**Conclusion:**

Understanding the paradox of access problems allows for different targets for change and different solutions to free up capacity in general practice to address the unmet need in the population.

## Introduction

Access to general practice is an important issue and the most relevant aspect of health care to addressing population health inequalities.^1^ In the UK, policies addressing access have favoured a simplified view of access, which focuses on the timeliness of appointments, rather than taking a broader view of the concept.^2^ The focus on speed of access has undermined other important aspects of care, such as continuity, despite the fact that continuity is valued by clinicians and patients,^3^^,^^4^ and is associated with several important outcomes such as reduced mortality,^5^ accident and emergency (A&E) use,^6^ and hospital admissions.^7^ General practice in the UK has been facing a growing workload and workforce crisis.^8^ Efforts to increase the number of GPs are inadequate,^9^^,^^10^ and skill mix-based solutions require considerable time and effort from GPs, so limiting their effectiveness.^11^ Meanwhile health inequalities in the UK population are growing.^12^ Past critiques of access to general practice policy in the UK recommended the application of existing theories of access in order to better understand and address persistent problems of access and health inequalities.^13^

This study aimed to understand the complexities of access problems to help develop relevant solutions that could increase workforce capacity and improve health inequalities. The research team applied Levesque *et al*’s conceptualisation of ‘patient-centred access to health care’,^14^ which juxtaposes five dimensions of accessibility of the healthcare system (approachability, acceptability, availability and accommodation, affordability, and appropriateness) with the abilities of patients to identify health needs and seek, reach, and utilise care. Building on this, the research team advanced the idea of access as ‘human fit’.^15^ Access as human fit builds on previous access research^16^^–^^18^ and other related theories about patient experience, including candidacy,^19^ in addition to the work of Levesque *et al*.^14^ It focuses on the interaction or fit between the abilities and needs of service users/population and the abilities and capacity of service providers/workforce/staff. The application of theory, combined with the approach and methods used, allowed for relevant perspectives to be understood in the context of their work and lives, and to be synthesised as interrelated patterns of issues. Importantly, understanding the paradoxical relationship between access issues can identify opportunities to free up capacity in the system to proactively address those in the population with unmet health needs.

**Table:** How this fits in

Access to general practice is an important topic, yet research and policies addressing access often use a simplistic definition, resulting in a lack of understanding of the complexities of longstanding interrelated problems. This study explains a paradox of access problems, in which the focus and attention on the increasing demand on general practice both creates and obscures another problem of unmet need. Reactive rules and policies to manage demand largely undermine continuity in favour of speed of access, and generate work that takes up capacity of staff and patients. Clinicians can, therefore, examine their current ways of working and identify ways to reverse the paradox to address hidden unmet needs and resulting health inequalities in the population.

## Method

This was a qualitative participatory case study of access to general practice in an area of northwest England, which has previously been described.^15^ It was a case study^20^ of access in what was at the time the geographic area covered by the Tameside and Glossop (T&G) Clinical Commissioning Group (CCG) (now divided between Greater Manchester and Derbyshire Integrated Care Boards). The area had a mixture of socioeconomic wealth and deprivation, as well as health inequalities across the population.^21^^,^^22^ There were a mixture of large and small practices, as well as both urban and rural settings. The core work of the project took place from 2015 to 2019, comprising lead author’s PhD research.

### Community-based participatory research

Consistent with the principles of community-based participatory research,^23^^,^^24^ a community-based research team (CBRT) was established early on in the project to share decisions in the design, execution, and dissemination of the work. This team consisted of 12 members of the T&G community and included patients, carers, GPs, practice staff, CCG staff, and members of the voluntary sector. The team met 35 times over 4.5 years of the project.

### Data collection

Data were collected between October 2015 and October 2016, and included transcripts and fieldnotes from 19 interviews,^25^ seven focus groups,^26^ and fieldnotes^27^ from 71 hours of observation^28^ in surgery reception areas and relevant health system and community meetings. Fifty four interview and focus group participants included 39 service users and 15 service providers. Service user participants ranged from 26 to 79 years of age and spoke of accessing care for those aged 0 to 101 years. Service user participants included those who possessed each of the nine protected characteristics of the UK Equality Act 2010 (age, disability, gender reassignment, marriage and civil partnership, pregnancy and maternity, race, religion or belief, sex, and sexual orientation), as well as those with various mental and physical health needs. Service provider participants had between 4 and 26 years of experience. Surgery observations spanned eight sites across the five areas in T&G that existed at the time, and included large and small practices. The data covered 36 of the 45 surgery and hub sites in T&G that existed at that time. Purposive sampling^26^ was used to seek a variety of contexts and perspectives.

### Data analysis

Analysis consisted of ongoing, inductive processing of the data using a modified framework approach,^29^^,^^30^ as well as abductive^31^ application of Levesque *et al*’s theory of access^14^ as the theory of human fit was developed.^15^ The analysis process began as data were collected, directing purposive sampling, and continued until the end of the project. The complexity and interrelatedness of access problems were understood through coding and mapping the data, aided by NVivo (version 11),^32^ and reflecting on the overall meaning of access and the entirety of the dataset, in partnership with the community-based research team, over time.

## Results

**Figure 1.**
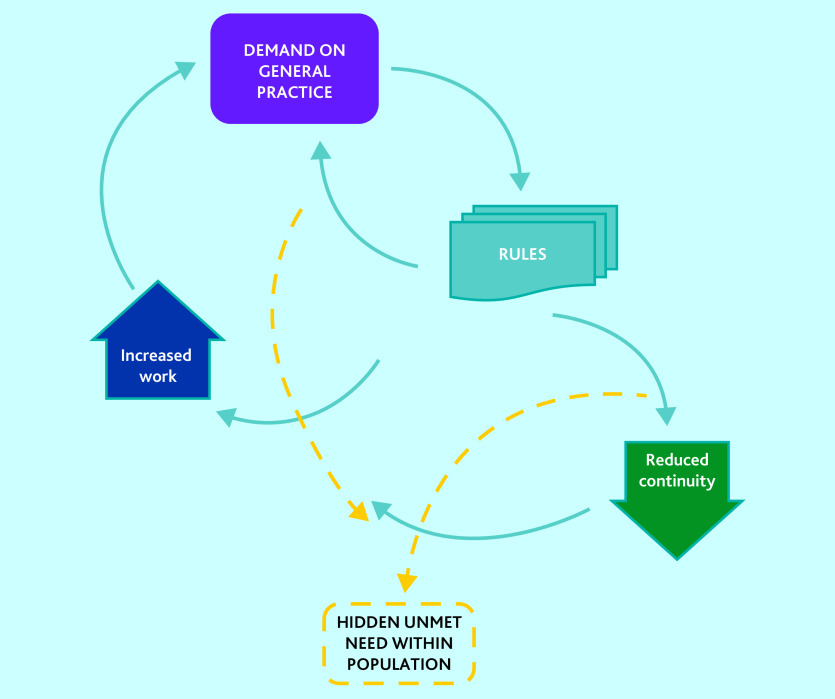
A paradox of access problems

This study contributes a novel description of access problems as a paradox of demand and unmet need. Figure 1 and the data below demonstrate how the real and perceived demand on general practice has led to a reaction involving rigid rules that undermine continuity and increase work. This fuels ongoing excessive demand as problems are not adequately addressed, while at the same time causes a different problem of unmet need. Unmet need consists of all of the health problems that go unrecognised or unaddressed by those in the population unable to seek care successfully. The unmet need arises out of the fact that general practices do not have the capacity or capability to be either flexible or proactive, in part because of the rigid rules imposed to limit demand, for some of the most ill and at-risk people in the population. It is these groups who experience persistent health inequalities. The demand problem not only causes unmet need through the mechanisms described, but it also paradoxically obscures it, because of the focus on using rules to make demand manageable. Those needs that cannot be met in accordance with the rules become invisible, and energy is focused on the visible demand.

This paradox is illustrated below using data from the study, including quotes from interviews and focus groups, extracts from ethnographic fieldnotes, and vignettes derived from the data, which portray indicative scenarios. Data are labelled with a unique code from each source.

### Demand on general practice

The demand on general practice was the focus of much of the attention around access. Participants highlighted the multifactorial and multidimensional nature of the demand, including from forces within the system. While it was ‘real’ in the sense that there were increasing numbers of people wanting appointments in general practice, responders also highlighted aspects of societal and medical culture that contributed. The following text describes some of these aspects of the perceived demand on general practice from various participants.

Patients may see general practice as a safety net, with no limits on demand:

*‘As general practice in the UK, we are the safety net for everything, in that we never say “no”. We always say “yes” to everything. And that’s great because it provides that safety net and allows other processes to work and frees up resources elsewhere, but actually, as the service that always says “yes” to every challenge that’s presented to it, we are beginning to struggle. We are a creaking gate, and it’s not going to take much before we go under*.*’*

(Interview responder [IR]05, GP)

Some of the demand on general practice may be due to patients’ social isolation and a lack of general social support:

*‘*[Our GP] *said he was getting patients coming to him saying, “Can you get someone to come and change my light bulb?” Because they couldn’t do it … And there is a lot of stuff like that. They don’t know where else to go just for social stuff. And isolation and loneliness is rife*.*’*

(FG6R3 [focus group responder], patient/patient participation group [PPG] member)

Over-medicalisation is seen by some GPs to be a problem:

*‘That’s what really* [upsets me] *about medicine today … I did a visit the other day with the* [trainee]*, to a nursing home, and there’s twenty people sat there in chairs, non-verbal, slumped over, most of them on twenty medications. And it’s where did we go wrong? When did we decide to stop asking people what they wanted, and just start medicalising everyone? I think we need to really push on this fact that we’ve medicalised people. And in terms of access, I think we’re the problem. We’re the people that have asked these people to keep coming back. “You need this. You’ve got this.” Labelling people. And as soon as you label someone with something, that’s it.’*

(IR18, GP)

As illustrated by this GP, some of the problems with access may be due to paternalistic general practice, generating over-reliance on services and lack of patient confidence to self-manage:

*‘Some of it generated by the medical profession who, you know, historically likes to be relied on and to know the answers, and they are there. Stories that I hear from … fortunately not from patients at this practice … they are just not really involved in their care because it was a paternalistic approach. Obviously then, that’s going to come back to bite you when they need you to say whether a cough is okay and whether a temperature is okay, whether their runny nose is okay, and whether this minor muscle ache is okay, because you’ve taken on the responsibility and taken control on every other consultation*.*’*

(IR04, GP)

Health promotion campaigns can be effective at generating more concerns about health, which leads to an increase in patient visits to GPs:

*‘Yes, usually multiple messages, yes. That phrase “I thought I had better get it checked out” — I mean we hear it so often, and it’s the dominant discourse out there, “Symptoms been going on, better get it checked out.” … People don’t know how else to check it out, apart from to see their trusted GP. Actually, yes, it probably is the quickest, most efficient, most effective way of getting it checked out, but actually there aren’t enough of us to do all that checking*.*’*

(IR04, GP)

A shortage of staff makes demand difficult for GPs to manage:

*‘But we have a huge issue at the moment … recruitment and retention of general practitioners. Because now I think, the last count, it was mentioned to us by* [GP leader name] *this week … thirty-five full-time GPs down in Tameside. Ac*ross *GM* [Greater Manchester] *I think it’s two hundred and sixty, and that’s ridiculous. So if that’s our starting, thirty-five GPs down, our starting point, how the hell are we ever going to get back to a normal way of working?’*

(IR18, GP)

Policy changes have led to wider engagement of GPs, taking them away from clinical practice:

*‘I just thought it was a real shame when* [PCTs] *were pulled down and the CCGs set up in their place … But what that also did, was to take away a whole load of clinical coalface time. Suddenly, like in our practice, we were suddenly having to manage without* [GP name] *here for two days a week and* [other GP name] *here for a day a week. And that must have been multiplied up and down the land, with GPs suddenly not seeing patients any more*.*’*

(IR13, GP)

Excessive workload can lead to burnout, reducing staff efficiency and effectiveness, as described by this group of practice managers:

*‘And if nine sessions was nine sessions, if they could come in at nine o’clock … and go home at five-thirty, every GP on the planet would stick with that. Not an issue. But they’re in at half-past six, seven o’clock in the morning. They’re going home at eight or nine o’clock at night. They’ve all got remote access to dial in from home to check their bloods and everything … by the time they get holidays, they’re practically ready to crack*.*’*

(FG1R1, practice manager)

*‘Yes, they’re burning out, aren’t they? And you only have to look at the clinical system at times that administration is processed, and it can be sometimes … I’ve got one particular GP half-one, two o’clock in the morning*.*’*

(FG1R4, practice manager)

*‘Doing things, yeah*.*’*

(FG1R5, practice manager)

[Others agree]

*‘You know, and because you look at the time stamp that it’s come through on the system and you think, “Oh”*.*’*

(FG1R4, practice manager)

These data demonstrate the complex contributing factors around the growing real and perceived demand on general practice. This feeling of overwhelming demand was the focus of much attention, and how to handle the demand dominated discussion and action around access. In order to render the demand manageable, practices have responded by layering on ever-more complex rules surrounding the making of an appointment.

### Reactive, rigid rules

The rules relating to how an appointment could be made varied greatly between practices, but were rigidly enforced in each case. While each rule had a logic and purpose, which was generally understood by practice staff, it could seem impenetrable to patients. Explaining the rules during attempts at accessing care was a lengthy process, and the policy-driven focus on speed of access over continuity resulted in a lack of fit between patients’ requests and what the surgeries had to offer. This in turn could generate problems because patients used the system in ways that were not intended, as described by a practice manager:

*‘See, we operate a walk-in three mornings of a week, which on paper sounds wonderful that you’ve got open access guaranteed for those three mornings. However, it creates sometimes more problem than good … Unfortunately, the patients kind of learn your new system, and they circumnavigate it. So there’s one GP particularly everybody wants to see, so they’ll all rock up on a Wednesday. You can guarantee Wednesday they’re queuing out the door. In the past as well, try doing more book on the day, which the trouble is then you then haven’t got the book ahead. You’re sort of balancing, and it’s only a finite number of stuff, but whichever way you try and do, you’re almost then doing it at the expense of other types of appointments, and you never really quite get that balance right*.*’*

(FG1R5, practice manager)

The practice managers had tried different things and felt frustration that they never seemed to help. Rules tended to be quite rigidly enforced, with little ability for the receptionists to make any exceptions when people’s needs did not fit into what was offered. One particular issue that caused problems was the definition of ‘urgent’ and ‘routine’ appointments:

*‘That’s what we get, because we’ve changed our appointment contact times for urgents and routine. We’ve split that up, and it still doesn’t suit everybody, which we know we’re not going to suit everybody, and we do alternates and try*.*’*

(FG1R3, practice manager)

*‘We never seem to suit anybody though, do you?’*

(FG1R2, practice manager)

[All laugh and agree]

*‘You know, we have the urgent appointments available at eight o’clock in the morning because if you’ve been up all night, you want to see a doctor as soon as possible and you want that appointment. For the routines we say, “Ring after eleven o’clock.” “Well, I’m in work. Nobody else can ring up, and I don’t get an hour, and I work twelve hours a day.” And that’s all you get because they want their appointment to be given when they ring up, but the system doesn’t allow it*.*’*

(FG1R3, practice manager)

One patient explained how the rigidity of the rules could be a particular problem for patients who used the system infrequently:

*‘I go to* [GP Surgery 12] *in* [town]*. I don’t know a great deal about it because I don’t go very often. And I think sometimes that can go against you, you know, because then when I do have to go, I don’t know what the procedure is, so I consistently get it wrong. You know, you’re like, “You can’t ring up at this time for this, you can’t ring up …” So I think sometimes when you don’t use a practice very often, you’re at a disadvantage*.*’*

(FG2R6, patient/voluntary sector worker)

Vignette 1 depicts an experience where basic information about the practice had changed to the disadvantage of the patient and carer, and illustrates how the rules were rigidly enforced (Box 1).

**Box 1.** Vignette 1: rigid enforcement of rules and rule changes not communicated

Participant IR12’s partner became ill when they were abroad on holiday. The treating hospital wanted the patient to have a follow-up appointment with his GP booked in order to discharge him safely. However, his surgery only allowed patients to book on the day, so it was not possible to obtain an appointment ahead of discharge. When the couple returned home and rang the next morning to book, they learned that, unbeknown to them, the surgery had advanced the time that they opened the phone lines in the morning, and therefore all of the available appointments had already been booked. (IR12, patient/carer)

In multiple surgeries, receptionists were observed declining patients’ requests for appointments and often having to state or restate the rule(s) to explain that decision. Vignette 2 demonstrates what often happens during the morning rush for appointments (Box 2).

**Box 2.** Vignette 2: no, no, no

As I sat in the receptionists’ space, I watched them struggle to enforce some complicated rules around appointments on to the population seeking care. They had slightly different rules on different days of the week, with varying amounts of telephone triage appointments, bookable telephone calls, and in-person appointments. Most of the in-person appointments could only be booked ‘on the day,’ and those that could be booked in advance with a known GP were full for as far out as they were scheduled. At a certain point in the morning, I started to hear the receptionists tell so many people, ‘No, call back tomorrow’ that I began to count. Between 9:45 and 11:45 a.m., I heard them say it to at least 28 people on the phone and 4 in person at the front desk. One aspect of telling so many people to ‘call back tomorrow’ was that the receptionists were actually generating more work for themselves tomorrow, adding to tomorrow’s demand of any new patient requests that might be made. It was clear that this was standard procedure at this surgery, even though it caused the receptionists stress to have the feeling of having run out of appointments, and to face the response from patients who were not happy with that answer. Some of the patients had already been trying to get an appointment on previous day(s). There was no guarantee there would be enough appointments the next day. In fact, the opposite was probably more likely. Patients were also frustrated at having to wait on hold, only to get that answer. Receptionists expressed to me that they wished they could put on the message that patients listen to when on hold that there were no more appointments for the day, as that would save them having to tell patients who had waited. Some of the receptionists there, who lived locally, told of being harassed by patients, when they saw them on the bus, for example, about not being able to get an appointment at the surgery. The receptionists had little ability to make exceptions to the rules, though occasionally the GPs would. (Fieldnote extract S1901, S1902)

Interestingly, the same surgery had some days that operated on more of a triage system, and the receptionists reported that they preferred those days because their job was simpler:

*‘*[Receptionist name] *is showing me how on Monday the appointments are all telephone calls.’ ‘We like that, don’t we* [other receptionist name]*?’*

*‘Yeah’*

*‘Because it’s easy for us. We just book* the call. Because we can’t be making these decisions, and people think we are blocking, but when doctors say, “no,” there is nothing we can do.’

(Fieldnote extract S19O1 [site, observation])

The difficulties that staff experienced in explaining and enforcing rules tended to cause them stress and reduce their job satisfaction. This in turn drove staff turnover. While continuity of clinical staff has been extensively studied, this study found that continuity of reception and administrative staff is also important in managing access.

### Continuity: important but undermined

Many previous policies about access in general practice have tended to undermine continuity by prioritising rapid access above all else. This includes the very basic notion of whether a patient has ‘a doctor’ or whether they are registered with ‘the practice’:

*‘It wasn’t a practice when I first came here thirty years ago. You saw your actual doctor. Now it’s a group practice of doctors*.*’*

(IR12, patient/carer)

Patients found doctor turnover to be problematic, and the policy solution of requiring practices to specify a ‘named GP’ for every patient has had mixed success:

*‘Oh, I’m not criticising the locums, I’m just saying that people can be anxious if they’ve had a system where they’ve been able … they’ve known the doctors and the surgery for a long time, and then everything sort of disintegrates*.*’*

(FG2R8, patient/volunteer)

*‘I don’t see how it’s only the locum though, is it. I mean, I had this problem. My GP that I’d been seeing for years, he went. This is just before* [name GP surgery 45] *took over the one I go to* [name GP surgery 7]*. And since then, I’ve never had one doctor, I have a practice. If I’m now asked who my doctor is, I say it’s the* [name GP surgery 7] *full stop, you know*.*’*

(FG2R4, patient/volunteer)

*‘Yeah.’*

(FG2R5, patient/volunteer)

*‘Well, you’re allocated a name, but you don’t see that person*.*’*

(FG2R8, patient/volunteer)

*‘You’re right. And I’ve seen bits of paper from the hospital that says GP’s name, and it’s got this person, I think, “Who the hell’s he?”* [Laughs]*’*

(FG2R4, patient/volunteer)

The rules established to manage demand tended to undermine continuity, with a general assumption that any doctor should do:

*‘But to put it in perspective, well, from this surgery, from* [name GP surgery 23]*, I don’t feel we have a problem. Because there are three — one, two, three — yeah, three partners who are well established, they’ve become the favourites. So they* are more in demand. So … when someone brings up the fact that they can’t get an appointment with a doctor for how many weeks, in actual fact they can get an appointment with a doctor, they just can’t get an appointment with that particular doctor at that particular time. So to me that isn’t a problem.’

(FG6R1, patient/PPG member)

Continuity among other practice staff was also seen to be important. For example, staff who knew particular patients were able to use that knowledge to decide what care would suit them best. However, attempts to flex the rules to accommodate their needs usually generated additional work, because those exceptions were not planned for when making the rules.

### Increased unnecessary work

The enforcement and constant justification of the reactive rules created in response to demand took up considerable staff time and energy. The fact that the rules largely undermined continuity also contributed to the increased work because patients who wanted continuity tended to find ways to work around the rules. The fact that demand felt overwhelming led to ways of working that were inefficient, prioritising immediate closure of a contact episode in order to deal with the queue of waiting people rather than taking a little more time to sort out an issue definitively. How blood results were handled was a common example of this, as illustrated in Vignette 3 (Box 3).

**Box 3.** Vignette 3: lab work

Participant IR12’s 89-year-old mother had a blood test as requested by the surgery as routine for her condition/medication. When she rang for the results, she was told by the receptionist that they ‘weren’t happy with them’ and that she needed a repeat blood test and a urine sample. Following that, she called into the surgery to learn of those results, and the receptionist told her that she ‘needed to see a doctor’ but that her own doctor was away. The patient logically asked whether it could wait until that doctor was back and also a little longer because she herself was then going away. The receptionist did not know. The patient waited while she tried to find out, called back in later in the day, and still had no answer because the receptionist had not yet been able to ask one of the GPs the question. The patient informed her daughter of the situation and planned to follow up the next day to make the appointment. The daughter, who owns her own small business, told her mother of times she could attend the appointment with her within the next week if needed. She would hold those potential slots open until she heard back from her mother. The next day the reception staff rang the daughter to schedule the appointment for the patient. The daughter assumed her mother had been in and been told it could not wait. She was able to arrange it on the weekday that her business is closed, which she appreciated. Later that morning, the patient contacted her daughter to say the appointment was scheduled in several weeks, when both the doctor and patient were back. The patient had had a different conversation with a different receptionist that morning and scheduled a different appointment. So now they had two appointments scheduled and two different answers on the urgency of the result. They decided to keep the first and go to it to get some information about the result sooner rather than later. When they arrived for the appointment, the check-in screen told them it was with a locum who was running nearly half an hour late. When they got into the appointment, the locum asked how he could help, unaware that they had been asked to make the appointment to discuss an abnormal result. He checked her records and seemed confused. He did not see an abnormal test result that he would ‘lose sleep over’ and suggested that the patient go out to reception to ask for a telephone consultation with the person who asked for the test. He mentioned that they should try to have continuity in these cases when they could. The patient and her daughter eventually had a face-to-face appointment (the patient is hard of hearing so a telephone appointment would have been a challenge) with the patient’s own GP, who was largely unaware of the events that had happened since the first blood result, which it turns out was only mildly abnormal. It was not possible to inform the GP of all they have been through in the short time of the appointment, nor was it how the daughter wanted to spend the time when the main task was to understand the result. The daughter tried to convey some of it to the GP including that they did not know whether to be worried. This was met with an inquiry by the GP to the patient about why she would worry, in a way that felt to the patient and carer like the patient was being judged as a worrier, rather than acknowledging the knowledge gap that existed around understanding the significance of the result. (IR12, patient/carer)

The increased work created by the ways of working were not made visible within practice systems and were therefore largely unrecognised by staff. Importantly, the ways of working and rules reflect areas to target for change in order to optimise the fit of access and free up capacity for reaching out to the unmet need in the population.

### Unmet population need

The result of the processes described in the paradox so far means that there was little proactive care for those in the population with high health needs, but who, for various reasons, were not able to successfully reach care themselves. These groups include some of the frail older patients, patients with limited English, patients with mental health problems, carers, and working people. Some participants recognised that the unmet need was there, but that the current norm did not allow space to adequately address it. The issues experienced by some of these groups are described below.

Those who need care do not always engage with practice systems and rigid rules may make this worse, as this GP explained:

*‘Quite a lot of end of life and severely frail elderly people are not banging on your doors, tapping you emails, or ringing you up. They’re suffering in silence. So you absolutely have to create that space for someone, somewhere to look after them*.*’*

(IR13, GP)

Service users may not know about standard services that are available to them. This may be because in general practice they are not proactively offered a service in the same way as they are in other parts of the health system. This is illustrated by the lack of awareness of the availability of interpreters in primary care during a focus group discussion among patients and carers with limited English:

*‘Do you ever use an interpreter service? An interpreter for the appointments with your doctor? Anybody?’*

(Interviewer)

‘*No*.*’*

(FG3R1, patient/carer)

*‘No, not with the GP. The hospital, like urgent check-ups and things like that*.*’*

(FG3R3, patient/carer)

*‘Okay, at the hospital they provide an interpreter, but at the GP what happens?’*

(Interviewer)

*‘No one.’*

(FG3R1, patient/carer)

*‘No.* [Collective agreement from group]*’*

*‘I don’t think they … there’s nothing on the wall to say that, “We offer you an interpreter”. I’ve not seen anything. Whereas when you get a hospital letter you do, don’t you, it says, “If you require…”.’*

(FG3R9, interpreter/patient/carer)

One voluntary sector worker explains that vulnerable people may find systems difficult to navigate and tend to disengage:

*‘If you have a mental health issue … and you’re having to wait two weeks, that can make the difference between you going into crisis and ending up in A&E, or actually even taking an overdose or something worse … Most of the time … we’ll pick a phone up and say, “We’re from the* [name charity] *… and I’ve got a lady with me who’s not very well. You can’t see her for two weeks, and that’s not good enough. She can’t wait. She needs help now.” And we’ve never been refused an appointment on that day. So that’s normally what happens. It’s quite frustrating that the person making the phone call in the first place can’t get that success. And not everybody who hasn’t had that success would come to us. So I know personally that there are quite a few people who would just go, “Oh, it doesn’t matter.” And it can add to their low self-esteem.’*

(IR14, voluntary sector worker/patient/carer)

A focus group of carer/patients described how the systems and rules are often poorly adapted to carers’ needs:

*‘The things you’ve had to do … do you think you could have expected more support from your GP than you got?’*

(CBRT interviewer 1)

*‘Yes.’*

(FG4R3, carer/patient)

*‘Yes.’*

(FG4R1, former carer/patient)

*‘Yes, definitely.’*

(FG4R3, carer/patient)

*‘Yes.’*

(FG4R6, carer/patient)

*‘Okay, so you feel, even though the problems are related often to hospitals, that the GP could have been more supportive, and if you’ve approached the GP, or have you approached the GP to ask for more support? And if you have done, what’s been the reaction?’*

(CBRT interviewer 1)

*‘Well, it’s a waste of time doing that. You ring up for an appointment at the doctor’s, right, and the receptionist will say, “Oh, the doctor will ring you back.” So then you’ve got to wait. You’ve got to stop in and wait for the doctor to ring you back, which is exactly what happened to me yesterday … So then you’re waiting for a doctor to ring you back, and he decides then whether you’re not well enough* to come and see him. So you’ve got no chance of asking for support for looking after somebody.’

(FG4R6, carer/patient)

A discussion from a focus group of patient/carers demonstrates how negative experiences with systems and processes tend to discourage their future attempts to get support:

*‘They try to keep the visiting down as low as possible, I think, which they work hard, I know that.’*

(FG4R1, former carer/patient)

*‘You can understand.’*

(FG4R6, carer/patient)

*‘You can understand, but when you’re there, and you’ve been up all night for months, not just a week or a few days. It’s day in, day out, that’s how I was with* [husband’s name*]. You need some help, it’s hard.’*

(FG4R1, former carer/patient)

*‘You might have been really … I can well imagine worn down with the things that had happened. If you’d have gone to the doctor and just said, “Doctor, this is the problem”, the doctor would have somehow been able to support or assist you? Do you think that was possible?’*

(CBRT interviewer 1)

*‘No, I don’t.’*

(FG4R3, carer/patient)

*‘No.’*

(FG4R1, former carer/patient)

*‘No.’*

(FG4R2, carer/patient)

*‘It sounds like it doesn’t feel like that’s the kind of request that you could …?’*

(CBRT interviewer 1)

*‘No.’*

(FG4Rs [This refers to several members but not necessarily all. Multiple voices agreed ‘no’])

*‘So it isn’t the case you’ve actually made to have been rebuffed, you just don’t feel confident about going in the first place,* [Rs: no] *because of the experiences you’ve had?’*

(CBRT interviewer 1)

*‘Yes, yeah.’*

(FG4R3, carer/patient)

*‘Yeah.’*

(FG4R1, former carer/patient)

One participant, a former factory manager, explains how the rules and processes do not fit with some patients’ working lives:

*‘But even the making appointments was firstly stressful for them, but also it was very disruptive to work. Because they only phone between eight and half past. So they had to leave their machine. Well, a four person* [type of machine]*, that means the machine stops. And then they’re on the phone for quarter of an hour trying to get an answer, and that would go on for many days’ time … And so there were issues like that around appointments, as well as their ability to get one in a timely manner. It’s the arranging that has to be done in a timely manner as well.’*

(FG6R2, patient/PPG member)

One additional issue to highlight is that of patients who do not attend their appointments. They are often described as wasting resources, especially in the context of overwhelming demand. However, this study found that the phenomenon was more complex than that. When a patient does not attend for an appointment this can allow precious time for an overworked clinician to catch up if they are behind, perform some of the many other tasks required of them, or simply to take a short comfort break. The reasons that patients do not attend appointments are varied, with individual patients forgetting, a cancellation request not being processed, or a patient unable to get through to the practice to cancel. Patients who miss appointments repeatedly could be seen as those whose needs are not being met by the current system, and they may need flexibility in the rules to be able to reach care. Some practices totalled up the number of patients who did not attend appointments and posted them in the waiting room in an attempt to deter and shame those who regularly missed appointments. This study suggests that a more productive approach might be to explore what was not working for those people and modify systems to be more flexible.

Thus the data presented suggest that, not only do systems put in place to manage demand potentially act to increase that demand, but they also paradoxically increase the phenomenon of unmet need, while simultaneously hiding it from view because it is invisible to the systems and processes in place. A GP recognised this in a public meeting, explaining how current approaches to demand were not working:

*‘If you want to make a difference … look for the things you can’t see and at what makes us poorly in the first place … Mix of people in reception who will be seen in a given morning: x colds, x administrative help-like letter, 1 chest infection, few UTIs, etc. But, we need to do less for those people and more for those not there … Housing estate across street. Wouldn’t want to walk there alone at night. Over past decade: three suicides, one accidental child drowning, many premature deaths, lots of fights and fractures, horrific scene of a man who fell and died at home and some time later* [GP] *and police broke down door to find partially decomposing body.* [GP became visibly bothered remembering that and stated he hopes he never has to do anything like it again] *Numerous cases of child protective services. One woman they didn’t know about with a child with learning disabilities. We need a different response than what we are doing for better health in Tameside.’*

(Fieldnotes excerpt HW [healthwatch] 1)

This GP and others recognised that the hidden unmet need in the population required more attention and different action from general practice in order to reduce inequalities. Understanding the paradoxical effect of the focus on the visible demand is the first step towards finding the capacity in general practice to proactively address hidden unmet need.

## Discussion

### Summary

This study used participatory research, qualitative methodology, and applied theory to understand access problems in UK general practice from multiple relevant perspectives. As a result of the iterative analysis, problems of access are presented as a paradox in which the focus on and reaction to a real and perceived growing demand on general practice has led to layers of rigid rules to manage the demand, which have undermined continuity, created extra work for patients and staff, and, most importantly, contributed to a growing, but hidden, problem of unmet need for people who cannot, for various reasons, navigate the current system to reach appropriate care because there is little outreach or flexibility to help them. These people include some of the most at-risk groups in the population, such as carers, those with mental health problems, and people with limited English. Addressing the aspects of the paradox of access directly, by addressing unmet need, reducing unnecessary work, restoring continuity, and reducing and flexing rules, rather than focusing on demand, has the potential to address longstanding inequities of access to care.

### Strengths and limitations

Key strengths of this study are the participatory approach, qualitative methodology, and the application of theory. The participatory approach ensured that multiple perspectives were taken into account throughout the design and execution of the research. The community-based research team members contributed to successful recruitment of a variety of participants and sites, and they helped to decide what was important in the data during analysis. The qualitative methodology was well suited to obtaining context-rich data that allowed for understanding of complexities and underlying factors. The applied theory of access, as opposed to a simplistic definition, facilitated an awareness of relevant factors around human interactions and fit, which enabled the paradox to be understood and agreed by the diverse team.

Potential limitations include the single geographic area and the lack of quantitative data. However, the issues observed were not specific to T&G and the contextual richness of the qualitative data goes beyond quantitative data. Another potential limitation is the relevance of the data, collected and analysed before the COVID-19 pandemic. However, this description of the complicated state of access to general practice before the significant changes layered on during the pandemic is essential to understanding the deeper roots and origins of problems that still exist, and have been compounded.

### Comparison with existing literature

The contribution of the description of the paradox of access problems complements much of the current and existing literature. Longstanding access problems relating to health inequalities are still present,^12^^,^^33^^–^^35^ and the paradox represents an effort to shape the complexity of those interrelated problems into something that can be addressed, which is rooted in a strong theoretical foundation. In this way, the current study is a direct response for overdue calls for theory to be applied in addressing these issues in a different way than policy has done for decades.^13^ The findings also resonate with the growing body of literature that links continuity to better health outcomes.^5^^–^^7^

### Implications for research and practice

This study provides a foundational description of longstanding access problems in general practice before the COVID-19 pandemic. Access to general practice has continued to be a contentious issue during the pandemic,^36^^–^^39^ and further research can build on these findings to understand how rapid changes during the pandemic have compounded, or helped, the existing issues described. GPs and practice staff will likely relate to these findings, which largely consist of accounts and observation of their working lives. By understanding the paradox of access problems presented in this study, clinicians and staff have an opportunity to see if there are ways to shift the current focus from demand to unmet need in their practice, and, in the process, rethink rules where flexibility is needed, restore continuity, and reduce unnecessary work to free up capacity. As health inequalities have worsened in the pandemic, this is even more important for general practice. Finally, the paradox description provides policymakers with an alternative target for change to improve access. Importantly, it is not simply about more appointments, more GPs, or more alternative roles. It is about understanding the importance of human fit for all in the population, including those least able to recognise health needs and successfully seek care. General practice needs resources, incentives, and capacity to do work that reverses the paradox, to stop focusing on the visible demand, and to look deeper and act more proactively to address the hidden unmet need. This includes restoring continuity so that staff and patients have a knowledge and respect of one another, which can help patients to feel seen and understood, and staff members to feel appreciated. These changes could improve patient satisfaction and staff retention, which would further reverse the paradox through reducing the unnecessary work that comes with the existing lack of human fit.
